# Effects of Upper-Limit Water Temperatures on the Dispersal of the Asian Clam *Corbicula fluminea*

**DOI:** 10.1371/journal.pone.0046635

**Published:** 2012-10-02

**Authors:** Inês Correia Rosa, Joana Luísa Pereira, Raquel Costa, Fernando Gonçalves, Robert Prezant

**Affiliations:** 1 Department of Biology, University of Aveiro and CESAM (Centre for Environmental and Marine Studies), Aveiro, Portugal; 2 CIEPQPF, Department of Chemical Engineering, University of Coimbra, Coimbra, Portugal; 3 College of Science and Mathematics, Montclair State University, Montclair, New Jersey, United States of America; University of Western Australia, Australia

## Abstract

Temperature is a determinant environmental variable in metabolic rates of organisms ultimately influencing important physiological and behavioural features. Stressful conditions such as increasing temperature, particularly within high ranges occurring in the summer, have been suggested to induce flotation behaviour in *Corbicula fluminea* which may be important in dispersal of this invasive species. However, there has been no experimental evidence supporting this hypothesis. It was already proven that *C. fluminea* drift is supported by a mucilaginous drogue line produced by mucocytes present in the ctenidia. Detailed microscopic examination of changes in these cells and quantification of clam flotation following one, two and three weeks of exposure to 22, 25 and 30°C was carried out so that the effects of increasing water temperatures in dispersal patterns could be discussed. Results show that changes in temperature triggered an acceleration of the mucocytes production and stimulated flotation behaviour, especially following one week of exposure. Dilution of these effects occurred following longer exposure periods. It is possible that these bivalves perceive changing temperature as a stress and respond accordingly in the short-term, and then acclimate to the new environmental conditions. The response patterns suggest that increasing water temperatures could stimulate *C. fluminea* population expansion.

## Introduction

Over the past decades there has been a growing interest in studying biological responses of aquatic organisms to increasing temperature. This interest is linked to attempts to envision effects of climate change with the expected increase in water temperature [e.g. 1,2]. In addition to issues related to climate change, other human activities, such as the growing investment in the construction of reservoirs and canals and the consequent change of the thermal regimes of altered systems, contribute to the interest on the effect of temperature in biological responses [3]. Temperature is a decisive environmental variable that greatly determines metabolic rates of organisms [4]. This is even more critical in animals exhibiting poikilothermic ectothermy, such as most invertebrates [5]. In the specific case of aquatic invertebrates, it has been proven that water temperature indeed influences important biological features, including feeding and digestion e.g. [6] and reproduction [e.g. 7,8].

The metabolic changes associated with temperature regimes are likely to translate into variations in population fitness and ultimately, at a macro-ecological scale, species distribution. In fact, the spatial distribution and dispersal patterns of a given species is often bound by temperature and thermal tolerance ranges and physiological optima [9]. Since thermal regimes and temperature changes can influence species distribution, a fundamental interest has been growing in correlating this information with invasive species. Understanding the physiological thermal range of an invasive species in turn can help us to understand their current and potential dispersal ranges and patterns. Such knowledge can also assist in predicting invasive potential of a new habitat and consequently support the design and implementation of preventive measures that can avoid or reduce the negative outcomes of the invader’s establishment. This perspective attains even higher relevance when applied to biofouling invaders, e.g. some freshwater bivalves. Invasive bivalves can accumulate in artificial low-flow areas (e.g. pipes and filters), adding negative economic impacts to the freshwater-dependent industry [10], [11]. In addition, some of these industries use heat treatment as a highly efficient enhancement agent in pest control either alone or in combination with chemical treatment [12], [13]. It is worth investigating how heated water relates to dispersal patterns adjacent (see e.g. the water temperature increase in the vicinities of thermal power plants as suggested by French and Schloesser [14]) and within the facilities to better understand the potential role of human-induced thermal change on the effective dispersal prowess of these highly migratory species.

An important biological invader that concomitantly is a relevant industrial biofouler is the freshwater bivalve *Corbicula fluminea* (Müller, 1774), commonly known as the Asian clam [10], [15]. This species is native to Southeast Asia, but underwent a massive global range expansion over the last century [16]. The specific Asian clam life-cycle features contribute to its success as an invasive species. Besides showing rapid growth rates and a short life span [17], this is an early-maturing hermaphroditic species that can self-fertilise [18] delivering high fecundity rates (up to 600–700 juveniles/day; [19]); offspring are generally released as pediveligers with about 230 µm length weighing as low as 10 µg dw [17]. Its introduction in non-native habitats and subsequent dispersal have also been frequently attributed to human activities, e.g. channelization, navigational dredging, commercial and recreational boating, food sourcing, marketing as fish bait [20], [21], [22]. Other authors suggest dispersal also through passive transport by waterfowls, attached to feet or feathers [21], or carried in fish gastrointestinal tracts [23]. However, there is little actual evidence supporting these non-human transport routes as primary means of dispersal [21], [23], [24]. Additionally, both downstream and upstream dispersal were claimed to occur [25]. If downstream transport with the water flow seems very likely to occur particularly in earlier life-stages due to the pediveligers and juveniles with reduced size and weight, upstream dispersion seems less obvious.

The discovery of the Asian clam’s mucilaginous drogue line [26] suggests the mucous-assisted flotation behavior to be an important mode of dispersal. The authors showed that long mucous drogue lines are produced by modified cells (mucocytes) packed along the inner demibranchs of the ctenidia at least in juvenile and small adult clams (up to 14 mm shell length). These drogue lines, together with other behavioral mechanisms, assist drift of the clam through flotation in response to water currents [26]. Stressful conditions, including increased water temperature, were already suggested to induce stochastic entrainment and dispersal or flotation behavior by the Asian clam [24], [26]. However, to date, no experimental evidences have confirmed this hypothesised relationship. Indeed, increasing temperature affects several fitness parameters on *C. fluminea,* including body mass, shell length, mortality and reproduction rate [27], [28]. Moreover, temperature seems to influence the activity of biological processes in other bivalves that assist attachment via byssus to previously unoccupied surfaces, thus promoting colonisation of novel habitats and consequently the species spread. For example, this is the case in *Mytilus edulis* [29], *Dreissena polymorpha* [30], [31], *Sinonovacula constricta* [32], and *Modiolus philippinarum* [33]. Therefore, it is possible that temperature plays a role in the dispersal mechanisms at a physiological level in other bivalves such as the Asian clam through changes in cellular structures that also promote dispersal.

This study was motivated by the identified gap in the literature on the effects of temperature over the physiological basis of the Asian clam’s dispersal patterns. Specifically, this study assesses the effects of increasing water temperature in the production of the mucous drogue line by *C. fluminea*, as well as examines whether temperature-induced physiological changes actually translate into variations in the flotation behavior. The information reported and discussed here constitute fundamental knowledge that can be used (i) as a proxy of the effects of global climate change in the dispersal patterns of this invasive bivalve, and (ii) as a starting point towards the development of more efficient temperature-dependent control methods in freshwater-dependent industries affected by the Asian clam.

## Materials and Methods

### *Corbicula fluminea* Collection and Maintenance

During early spring 2011, clams (shell length below 11 mm) were collected from Six Mile Run, a shallow creek located in Somerset County, New Jersey, USA (N40°28.166’/W74°32.620’). At the moment of the clam’s collection the water temperature was 10.1°C, with pH of 8.73. The creek has a well-established *Corbicula fluminea* population with densities of about 400 individuals m^−2^ with a mean shell length of 16.43 mm; the dominant substratum is coarse sand with 2.42% organic content, and turbidity, dissolved oxygen and EPT (Ephemeroptera-Plecoptera-Trichoptera) index here were recorded at 3.8, 8.7 mg L^−1^ and 3, respectively [34].

Clams were transported to Montclair State University in plastic buckets filled with creek water. Four clams were immediately sacrificed and treated as histological samples (see the histology section below for details) to provide a field reference in data analysis. All other clams were acclimated for 1 week in glass aquaria: twelve glass aquaria holding 16 clams, each animal inside a crystallizing dish (10.5 cm diameter, 4.5 cm height) placed at the bottom of each aquarium; the aquaria were filled with 75 L field water, which was gradually replaced with dechlorinated tap water along the acclimation period. An externally driven recirculation and filtration system was set in the aquaria to ensure water quality during the acclimation period and the temperature in the culturing room was kept at 22±1°C with a controlled photoperiod of 16h^L^:8h^D^. An equivalent set of animals was acclimated following the same protocol, the differences being that each crystallizing dish contained a bottom sediment layer (ca. 2 cm thickness of coarse sand brought from the field). These clams were further used for a parallel assessment of flotation behavior (see below for details). The crystallizing dishes allowed the necessary moving of the clams before the observation periods along the experiments with minimal disturbance, so that records on behavioral parameters relate only to the established stimulus of increased water currents (see below for details) rather than reflect effects of direct animal handling.

An additional water sample was collected in the field to serve as a microalgae inoculum grown in the laboratory to feed the clams. The water sample was acclimated for 5 days in the laboratory and 1 L was then added to a sterilised Erlenmeyer vessel filled with 1 L of Mauro’s 3M culture medium [35]. The microalgae were left to grow under permanent aeration, continuous illumination at 22°C, for 10 days.

### Exposure Conditions

Clams were exposed to different temperatures in aquaria filled with 75 L dechlorinated tap water. The same aquaria and crystallizing dishes used in acclimation were used in experiments in order to avoid additional stress on the clams. The test aquaria were kept under a water recirculation system as previously described maintenance and water volume was kept constant in the aquaria along the exposure period by periodic re-filling with dechlorinated tap water as necessary. The photoperiod was maintained in 16h^L^:8h^D^ and each aquarium was added with a common heater to establish three independent temperature treatments of 20, 25 and 30°C with 3 replicates each. The tested temperatures are within the tolerance limits described for the Asian clam (upper temperature limit: 36–37°C) [36] and reflect actual variations in the field at the site where the clams were collected, which has been regularly monitored (R. Prezant, personal communication; US Geological Survey database - http://waterdata.usgs.gov/nj/nwis/current/?type=qw&group_key=basin_cd, at Mill Brook at Route 10 or Pequannock River at Oak Ridge), as well as summer conditions in other invaded systems [e.g. [37]–[39]. Additionally these temperature ranges can be found especially in the vicinities of power plants using cooling waters from abutting streams and discharging thermal effluents [20], [40]. Temperature was recorded four to five times daily throughout the exposure period for monitoring purposes. The 16 clams in the crystallizing dish placed at the bottom of the aquarium constituted each experimental unit. To control potential interference of size-dependent effects in the results, clams were grouped according to established size classes so that each replicate contained 8 clams less than 5 mm, 4 clams 5–8 mm and 4 clams 8–11 mm shell length. Larger clams were excluded from the experimental setup since production of the mucous drogue line has, to date, only been documented for clams smaller than 14 mm shell length [26]. Unlimited food resources were provided to the clams by periodic infusion of microalgae into aquaria at least twice per week (see previous section).

### Histological Procedures and Microscopic Analysis

Microscopic inspection of ctenidial mucocytes distributed in the inner demibranchs of the experimental clams was performed. Samples composed of 4 clams (2 with less than 5 mm, 1 with 5–8 mm and 1 with 8–11 mm shell length) were brought from the field (see above for details) and taken from each replicated crystallizing dish following each exposure period (one, two and three weeks of exposure to different temperatures). Clams were opened through gentle forcing between valves with a blunt needle and their soft tissues (whole body) were fixed in Zenker’s fluid overnight. The fixed samples were washed overnight in running tap water and dehydrated through an increasing concentration series of ethanol and toluene solutions. After dehydration, tissues were carefully embedded in paraffin wax (Paraplast©, 60°C m.p.) so that antero-posterior sections could be made in the organisms corresponding to longitudinal cuts of the demibranchs. Blocs were sectioned using a microtome set to 5–7 µm cut thickness. Sections were stained with sodium borate buffered aqueous toluidine blue.

Slides containing demibranchs of each individual were located and the first 9 sequential sections were selected for further analysis. Light micrographs were taken from the best cut within each ribbon using an electronic image acquisition system (Olympus SC30 digital camera; analySIS getIT) coupled to an Olympus CKX41 inverted microscope. In order to reduce the potential interference in the results of intra-demibranch spatial variability, the observed demibranch images were artificially divided into three sections and mucocyte size and number were assessed within each section. A linear segment of ca. 970 µm was established over each section following a longitudinal direction across the ctenidial lumen and all mucocytes located within this distance were counted. Data yield from this analysis were expressed as number of mucocytes per µm. Five mucocytes were randomly measured within each section thus 15 measured cells per individual accounted for the dataset. Micrographs were analysed using the open-source software ImageJ, Image Processing and Analysis in Java (available at http://rsbweb.nih.gov/ij/index.html; accessed Jan 2012).

### Flotation Behaviour

Clam flotation behavior was observed following one, two and three weeks of exposure to different temperatures. Four observation periods of 10 minutes each were made during the last day of each defined exposure period and before collecting clams for histological procedures. Following the approach by [26], each replicated crystallizing dish containing the clams was re-positioned in the test aquaria under a gentle water current produced by the aquarium filtration system (maximum flow rate of 13.25 L min^−1^) to stimulate clam flotation. When submitted to the water flow, the clams would distend their exhalant siphon and foot and gently lifting off the substratum, and would drift into the water column. Such an integrated sequence of movements occurring within each 10-min observation period was recorded as a drift. Data were expressed as relative frequency of drifts observed in each replicate per day. Given the infaunal character of this bivalve species and provided the suggested association between stressful conditions and drifting, we hypothesised that sediment could play a protective role hence inhibiting flotation. Thus, the exposure and drifting record protocol was repeated using clams contained in the crystallizing dishes added with a bottom sediment layer (ca. 2 cm thickness of coarse sand brought from the field).

### Statistical Analysis

Mucocyte size and number were addressed as independent experimental variables. Mean values within individuals that composed each replicate were calculated and used to further determine the product between size and number as an additional variable to complement the analysis. The mean values between individuals composing each replicate were used in the statistical analysis (n = 3). Detailed graphical exploration of the datasets was run following Zuur et al. [41] and Quinn and Keough [42] to check the assumptions required for parametric statistics. Slight deviations were found mostly to normality but occasionally also to homocedasticity, and data transformation was applied for improvement whenever necessary.

A repeated-measures (RM) ANOVA approach was followed for each histological variable considering temperature as the between-subjects factor and time (week) as the within-subjects factor. Whenever a significant interaction between time and temperature was present, simple main effects of temperature or week were analyzed separately [42] as follows. The main effect of temperature was addressed by running one-way ANOVA using the within-cells error term calculated from the former RM ANOVA output as denominator of the test F; the same applied to the main effect of time (week) but using rather one-way RM ANOVA with week computed as a within-subjects factor and using the overall (within subjects) error term as denominator of the test F. Whenever deviations from sphericity were found as measured by ε, the degrees of freedom were adjusted in the RM ANOVA procedures using the most conservative Greenhouse-Geisser estimate [42]. A Tukey *post-hoc* test followed whenever applicable to determine significant differences between treatments of time or temperature. The Bonferroni procedure was used to conservatively adjust the associate significance level of the family-wise type I error. A total of three comparisons applied for each variable (three temperature or three exposure periods) thus a significance level of 0.017 was used in the analyses. Explorative Pearson correlation analysis was run to assess whether mucocytes size is related to cell number within each combination of timepoint and temperature.

Clam flotation behavior was quantified as the ratio of the number of recorded drifts by total number of clams (drift ratio) within each replicate at each observation timepoint (last day of the first, second and third week of exposure). Differences between temperature treatments were assessed through a RM ANOVA approach using observation time (week) as the within-subjects factor and temperature as the between-subjects factor. The sphericity assumption, as well as simple main effects of temperature or week, was addressed as described above. Explorative Pearson correlation analysis to assess whether each of the mucocyte variables related to drifting behavior as temperature changed within each exposure period was also performed.

## Results

Temperature was the main factor tested for its influence on the production of the ctenidial mucillagineous threads by the Asian clam *Corbicula fluminea*. Low variation in temperature was generally found within treatments along the test period. The test organisms were exposed to three temperature treatments set to 20, 25 and 30°C. Appreciable deviation of the lowest controlled temperature to the set point was observed: 22±1.1°C (mean ± standard deviation). The actual temperatures measured for the other two treatments were 25±0.5°C and 30±0.9°C. Because of the noticed difference between the 20°C set temperature and the corresponding mean temperature monitored during the exposure, the lowest temperature treatment is henceforth considered to be of 22°C. No mortality was recorded during the test period in any experimental treatment.

**Figure 1:**
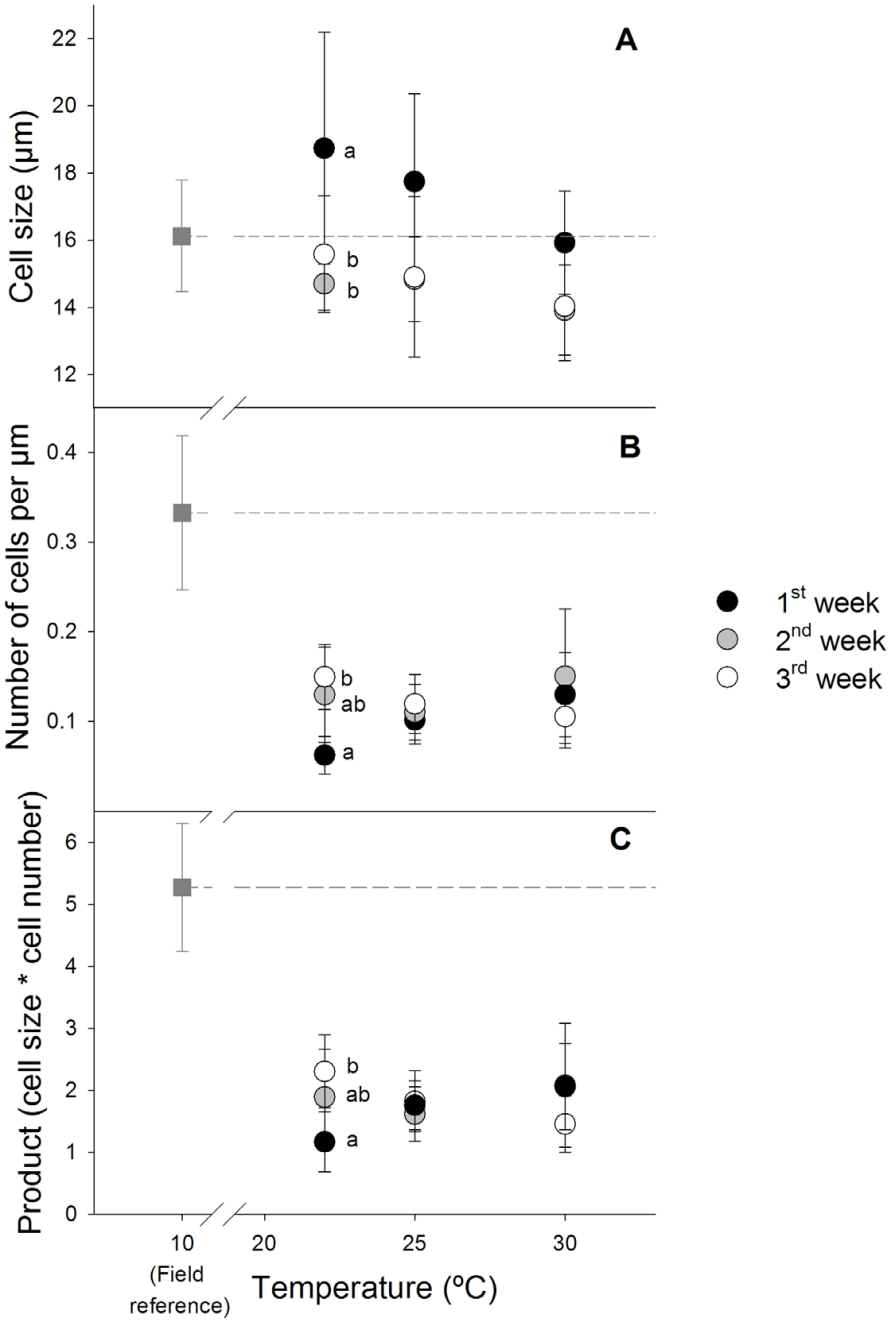
Variation of ctenidial mucocytes in clams exposed for one, two, and three weeks to different temperatures. (**A**) Mean mucocyte size; (**B**) Mean mucocyte number; (**C**) Product between corresponding mucocyte size and number. Field reference corresponds to untreated clams and is represented by the square grey mark and dashed line. Letters placed next to points denote differences between groups of exposure period at each exposure temperature following the *post-hoc* Tukey test. Error bars represent standard deviation.

The graphical trends in figure 1, representing the variation of ctenidial mucocytes in clams exposed to 22, 25 and 30°C for distinct exposure periods (one, two and three weeks of exposure), suggest that exposure time has an important role in modelling the response of ctenidial mucocytes to increasing temperature. This pattern was confirmed by the significant interaction between temperature and week found for cell number and the product between cell number and cell size (Table 1). Main time-dependent effects were observed, with special emphasis on ctenidial mucocytes size. Significantly larger cells were found following the first week of exposure to all temperatures as compared to the records obtained following two and three weeks of exposure, with changes statistically significant for 22°C (Fig. 1.A; Table 2.A). This pattern remained unchanged as temperature increased, which is consistent with the lack of significant differences in mucocyte size of clams exposed to different temperatures within each exposure period (Fig. 1.A; Table 2.B). Cell number was also significantly affected by the exposure duration, at 22°C. At this temperature, significantly fewer mucous cells were found in clams following the first week of exposure as compared to the third week of the test (Fig. 1.B; Table 2.A). Cell size was multiplied by the corresponding cell number to produce a new variable for analysis. To some extent this additional variable represents normalized changes in mucocytes assisting the proper interpretation of the main variables (cell size and cell number). For example, an increase in cell number may only mean that cells divided if accompanied by the proportional reduction in cell size. The product between cell size and number following one week of exposure to 22°C was significantly lower than that recorded following three weeks of exposure similarly to the observed pattern for the number of mucocytes (Fig. 1.C; Table 2.A).

**Table 1:** Summary of repeated measures ANOVA applied to address the effect of time and temperature in mean ctenidial mucocytes size and number, as well as corresponding product (mucocyte size x number). Significant effects (α = 0.05) are marked in bold.

	Cell size				Cell number				Product (number*size)			
	df	MS	F	p	df	MS	F	p	df	MS	F	p
**Within-subjects**												
Week	2	25.786	29.30	**<0.001**	2	0.003	5.30	**0.022**	2	0.172	0.177	0.212
Week*Temperature	4	0.904	1.027	0.432	4	0.002	4.49	**0.019**	4	0.602	6.204	**0.006**
Residual	12	0.880			12	0.001			12	0.097		
**Between-subjects**												
Temperature	2	7.564	7.64	**0.022**	2	0.001	2.15	0.198	2	0.105	0.958	0.435
Residual	6	0.990			6				6			

**Table 2:** Summary of (A) repeated measures ANOVA and (B) one-way ANOVA applied to address the main effects of time or temperature, respectively, in mean ctenidial mucocytes size and number, as well as corresponding product (mucocyte size x number).

(A)	22°C				25°C				30°C			
	df	MS	F	p	df	MS	F	p	df	MS	F	p
**Cell size**												
Week	2	13.903	17.62	**0.010**	1.1	16.04	7.00	0.104	1	9.136	8.34	0.102
Residual	4	0.789			2.3	2.29			2	1.095		
**Cell number**												
Week	2	0.007	7.00	**0.010**	1.4	0	0	1.000	1.2	0.001	1.00	0.337
Residual	12	0.001			12	0.001			12	0.001		
Product (cell number*cell size)												
Week	2	1.115	11.49	**0.002**	1.9	0.036	0.37	0.554	1.3	0.348	3.59	0.083
Residual	12	0.097			12	0.097			12	0.097		
**(B)**	**Week #1**				**Week #2**				**Week #3**			
	**df**	**MS**	**F**	**p**	**df**	**MS**	**F**	**p**	**df**	**MS**	**F**	**p**
**Cell size**												
Temp	2	6.077	3.91	0.082	2	0.709	3.30	0.108	2	2.587	2.64	0.151
Residual	6	1.554			6	0.215			6	0.981		
**Cell number**												
Temp	2	0.004	2.05	0.210	2	0.001	0.65	0.555	2	0.001	0.50	0.630
Residual	6	0.002			6	0.002			6	0.002		
Product (cell number*cell size)												
Temp	2	0.771	1.77	0.250	2	0.166	0.381	0.699	2	0.372	0.853	0.472
Residual	6	0.436			6	0.436			6	0.436		

Significant effects (α = 0.017) are marked in bold.

Figure 1 and Table 1 (see cell size in particular) suggest temperature-dependent effects within exposure period. However, these were not statistically significant as main effects were analyzed even within the first week of the test, where the most consistent changes could be noticed with temperature (Table 2.B). Nevertheless, mucocyte size decreased consistently while the number and product increased consistently with increasing temperature following one week of exposure (Fig. 1.A). Other general graphical trends could not be statistically confirmed, including the record of fewer mucocytes and lower product between cell size and number with increasing temperatures following three weeks of exposure (Fig. 1.B and 1.C).

An inversely proportional relationship between cell size and cell number is clear following one week of exposure to increasing temperatures, with more but smaller cells being produced (Fig. 1.B as compared to 1.A). This is supported by a significant negative correlation between the number of mucocytes cells and their size (Pearson coefficient  =  −0.442; p = 0.016). However, this pattern was not confirmed through time, with non-significant correlations being found following two and three weeks of exposure (Pearson coefficient  =  −0.201, p = 0.271; and Pearson coefficient  = 0.020, p = 0.920, respectively). Furthermore, the graphical pattern of the product between cell size and number (Fig. 1.C) seems to resemble that shown for cell number (Fig. 1.A) rather than provide an intermediate picture between the potentially related endpoints.

The ctenidial mucocytes were also examined immediately after clam collection from the field in order to provide a reference value for untreated organisms (Fig. 1; “field reference”). Although cell size in exposed clams is within the same range of that recorded for field clams, treated clams had fewer cells showing similar size to those of field clams (Fig. 1.B as compared to Fig. 1.A). The duration of exposure plays an important role on how temperature drives the changes relatively to the field reference if this is taken as a surrogate for the basal stage of the organisms. Indeed, following one week of exposure, all endpoints tended to converge to the corresponding field record as temperature increases, while following three weeks of exposure the opposite occurred. This contrasts with an increasing distance from the unexposed reference to the records in clams exposed for three weeks as temperature rises.

**Figure 2:**
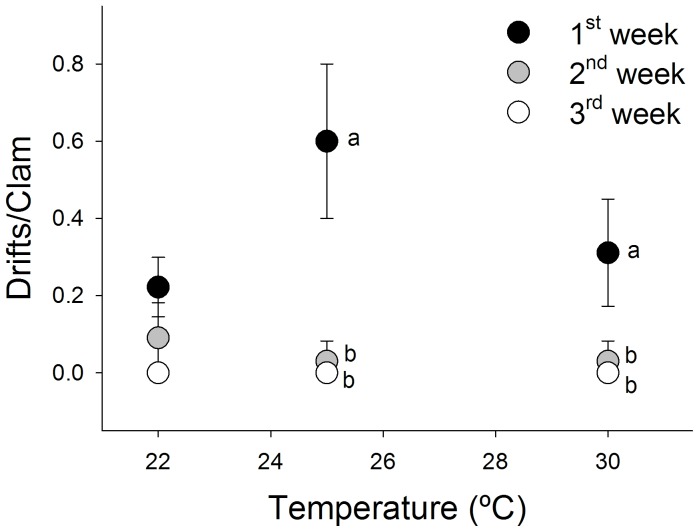
Mean clam flotation following exposure to different temperatures for one, two and three weeks. Letters placed next to points denote differences between groups of exposure period at each exposure temperature following the *post-hoc* Tukey test. Error bars represent standard deviation.

Time was the main factor explaining the variation in the clams’ drifting behavior (Table 3) with the highest proportion of drift-responsive clams being observed in the first week of testing (Fig. 2). Following the first week of exposure there were significantly more drifts than following two and three weeks of exposure (Fig. 2; Table 4), at the higher temperature treatments of 25 and 30°C (Fig. 2; Table 4). Following two weeks of exposure to any of the tested temperatures clam flotation behavior was very rarely observed and following three weeks of exposure clams did not drift at all. The slight differences in the drift records between these two latter time-points were not confirmed statistically (Fig. 2; Table 4). In these experiments, temperature did not statistically affect drifting behavior (Table 4), although following the first week the clams flotation behavior clearly increased as the 22°C compares to 25°C (Fig. 2). The parallel experiment run with clams that were given sediment protection yielded very definitive results. No drift was observed in any of the temperature treatments regardless the exposure period considered.

**Table 3:** Repeated-measures ANOVA referring to mean clam flotation following one, two and three weeks of exposure to different temperatures.

Source of variation	df	MS	F	P
Within-subjects				
Week	2	0.379	51.757	**<0.001**
Week*Temperature	4	0.045	6.146	**0.006**
Error	12	0.007		
Between-subjects				
Temperature	2	0.31	2.635	0.151
Error	6	0.12		

Significant effects (α = 0.05) are marked in bold.

**Table 4:** Summary of (A) repeated measures and (B) one-way and ANOVA applied to address the main effects of time and temperature, respectively, in mean clam flotation.

(A)	22°C				25°C				30°C			
	df	MS	F	p	df	MS	F	p	df	MS	F	p
Week	2	0.037	5.28	0.023	1.1	0.623	89	**<0.001**	1	0.173	24.71	**<0.001**
Error	12	0.007			12	0.007			12	0.007		
**(B)**	**Week#1**				**Week#2**							
	**df**	**MS**	**F**	**p**	**df**	**MS**	**F**	**p**				
Temperature	2	0.117	0.88	0.462	2	0.004	0.03	0.970				
Error	6	0.133			6	0.133						

Significant effects (α = 0.017) are marked in bold.

Correlation analysis to assess whether each of the mucocyte-related variables could be linked to drifting behavior revealed no significant associations (data not shown).

## Discussion

Bivalves are able to acclimatize to cope with changing environmental conditions or minimally show variation in macrostructural or microstructural form in response to these changes, e.g. in shell morphology [43], [44], behavioural traits, such as predator avoidance strategies [45], or valve movements [46]. There are studies reporting a wide-range in shell plasticity of populations of the Asian clam *Corbicula fluminea* inhabiting geographically adjacent habitats that show distinct environmental conditions [e.g. 47,48,49]. Other evidence of *C. fluminea* phenotypic plasticity is its proven ability to regulate body mass while facing long starvation periods as a final outcome of an efficient management of energy budgets and allocation to different body compartments [28]. This suggests that this bivalve has a large range for physiological adjustment to cope with potentially stressful conditions. The present study provides additional evidence on the ability of the Asian clam to adjust physiology and behavior as a response to changing environmental conditions. The results show that changes in water temperature, at least at high ranges (above 20°C), affect the production of the mucous drogue line by *C. fluminea* and its drifting behavior, thus possibly influencing dispersal patterns with consequences on population expansion. It should be noticed here that drifting by the Asian clam may not relate at least directly to physiological changes occurring in the ctenidial mucocytes. However, it should be kept in mind that changes with temperature were recorded consistently following the first week of exposure both in mucocyte cells and in flotation behavior. Although a relationship between the physiological and behavioral parameters was not confirmed statistically with the particular design employed in this study, this latter evidence seems to point towards an association between the production of the mucous drogue line and clam’s drifting driven by temperature.

Increasing water temperature, particularly to levels closer to the species upper tolerance limit (36–37°C [36]), can produce physiological stress. At less extreme conditions, increase in temperature generally relates to an increase in metabolic rates, particularly evident in ectotherms, with consequential changes in resources exploitation [1], [28], [50], [51]. The data recorded on ctenidial mucocytes in clams exposed to high-range temperatures seem consistent with this metabolic shift. In fact, increasing temperature apparently triggered an acceleration of the mucocytes life cycle thus favoring higher cell division rates [52]. As a consequence more but smaller cells were produced, especially after one week of exposure. On the other hand, the data collected on the reference sample (10°C) did not support this hypothesis as particularly evidenced by its higher number of mucocytes as compared to treated samples. This seems to indicate distinct mucocyte cycle patterns at low-range temperatures that would be worth to explore in future studies. Indeed, the literature is not consensual as to the effect of increasing temperature in mucocyte-like cells, making it even more speculative to advance with an explanation for the phenomenon. Studies on other secretory cells that produce mucin substances support our results for high-range temperatures, e.g. Gammert et al. [53] showed that the number of goblet cells of the nasal mucosa of rats increased with increasing temperature. Conversely, other studies demonstrate the opposite, e.g. in mucous cells of the epidermis of catfish [54]. Along with these reported inconsistencies, the present study provides contradictory results when considering shorter (1 week) *versus* longer exposure periods. Specifically, the pattern of increasing cell division rates with increasing temperatures was not evident for longer exposure periods. Thus it is likely that the observed changes in mucocytes relate to an immediate response to a perceived environmental stress rather than reflecting a longer term shift in metabolic rates.

Prezant and Chalermwat [26] suggested that the production of the mucous thread and consequent drifting capability could be more readily induced when clams face stressful conditions. *C. fluminea* drift as quantified in the present study reflected, in particular, in the clams’ response to induced stress after one week of exposure. This is supported by the effects of temperature treatments on mucocytes size and number following the first week of exposure as compared to longer exposure periods. It is possible that the first week of increasing temperature acted as a critical stress that, in turn, induces mucocyte production that could more readily “allow” mucous drogue line and ultimately flotation behavior. As the higher temperature exposure continues, individuals might have adjusted their metabolism to the new environmental conditions thus acclimatizing to the new thermal regime. When clams were supplied with a sediment layer, no drift was recorded throughout the exposure period. Apparently, at least in this current experiment, the sediment plays an important protection, perhaps stabilizing, role that seems to compensate for the stress induced by increased temperature. Provided the infaunal habit of *C. fluminea*, the type of sediment has been argued to represent a relevant constraint to the species distribution in natural systems such as creeks and rivers [e.g. 55]. However, the clam has also been reported to proliferate massively in underwater structures and channels of freshwater-dependent industries [10], hence studies such as the present addressing the species dispersal abilities in systems lacking sediment should not be disregarded. Since a direct relationship between mucocyte activity and drifting behavior was initially hypothesized, the role of sediment was only assessed using drift ratios as an endpoint. Further histological studies on the effects of temperature in mucous production in test systems supplied with sediment would help clarify the extent of its protective role, and ultimately the role of temperature as an actual stress agent.

It was already proven that *C. fluminea* mucous cells residing in the inner demibranchs produce the mucilagineous drogue line, which has an active role in the species’ dispersal ability [26]. In this way, and following the suggestions by McMahon [24] on the eventual effect of increasing temperature as a promoter of clam’s lift-off into the water column, it seems reasonable to assume that our results indicate that increasing temperature at high-ranges could enhance the Asian clam dispersal ability through the increased production of the mucous drogue line as an immediate response. Although this seems a reasonable prediction for scenarios where the water temperatures range within those tested here (22° to 30°C), it should be stressed out that the data obtained with the field reference (untreated clams facing 10°C water temperature) did not corroborate it, highlighting the need for future studies that address mucous production and drift behaviour under low-range temperature changes. Temperature also influences byssus production in other bivalves. For example, Young [29] and Clarke and McMahon [31] found that increasing temperature leads to increasing byssus thread number in *Mytilus edulis* and *Dreissena polymorpha*. While byssal thread and the mucous drogue line both play key roles in relevant species dispersal, these structures are ontogenetically, phylogenetically, and physiologically distinct: byssal threads form from a complex multiglandular byssal gland releasing a quinine tanned protein with a mucin and collagen component and emerge via a pedal ventral grove [56], [57]. Despite these differences, a behavioral parallel can be traced that suggests a link between temperature and dispersal potential in byssus-producing species and mucoid drogue line dispersing species.

With water temperature alteration, other water properties such as viscosity are affected. Increasing temperature causes decreased viscosity and can contribute to decreased buoyancy of floating particles. This could have consequences on bio-mechanical activities such as the drifting or swimming efficiency of aquatic organisms [1], [58]. Thus, decreased viscosity with increasing temperatures may eventually help explain the observed increase in flotation behavior between clams exposed to 22°C and those exposed to higher temperatures during the first week. However, a monotonic trend for drift intensification with increasing temperature could not be recorded. Other authors have not been able to demonstrate a straight relationship between changes in temperature and viscosity-driven changes in drift. For example, Wang and Xu [32] concluded that with decreasing temperature there is a more frequent drifting behavior in bivalve veliger larvae, and Williams [59] found that different invertebrate species showed no consistent drifting responses as temperature changed. This suggests that clam drifting (and equivalent behavior in other organisms) results from a complex interaction of several factors including physical parameters (e.g. viscosity), inherent physiology, and environmental challenges. Knowledge on how this complexity of factors enhances or inhibits dispersal is of obvious relevance when considering invasive species such as *C. fluminea*. Furthermore, it contributes to the understanding of the ecological dynamics of benthic communities, which is strongly influenced by invertebrate drift and settlement, either by the continuous loss of clams into the water column that reduces population density in a given location, or by its continuous settling out with important colonizing implications [60], [61].

The temperatures tested in this study are within the tolerance limits described for the Asian clam [see 36] and can also be found in the field especially in the vicinities of power plants using cooling waters from abutting streams and discharging thermal effluents [20]. Adding to the potential dispersal shifts induced by temperature changes that might occur in lotic and lentic habitats of Asian clam populations are the potential implications and consequences of global climate change, and the alteration of thermal regimes due to dam and canal construction. Thus, the results constitute a valuable add-on to the baseline information that is required to predict environmentally-driven changes in dispersal patterns of the Asian clam. It is also important to consider the fact that the higher efficiency in control methods relying in high water temperature applied for short periods may not be as straightforward as presumed as we consider that a concomitant stimulation of dispersal within affected areas can occur representing a drawback in the method. Further research still applies to clarify temperature-dependent effects on *C. fluminea* dispersal. For example, in an ongoing study, we recorded remarkable differences in the interlamellar epithelium (totally absent or incipient ctenidial mucocytes) of *C. fluminea* demibranchs in individuals from a population established in a Portuguese channel as compared to the Somerset, New Jersey, USA population studied in the current experiments. Genetic typing (RFLP analysis and mtCOI sequencing) revealed no clear distinction between this population and the Somerset population examined in the present study i.e. both exhibit the same haplotype. It is reasonable to hypothesize that local adaptation may constrain general conclusions on the influence of environmental change in the species dispersal patterns. Furthermore, some authors [e.g. 62,63] suggested that the mucous produced in the demibranchs may nourish the developing embryos and/or assist the release of juveniles out of the gills. To tease apart potential issues of seasonality or geographic localities or difference induced by localized populations, further studies are being conducted addressing seasonal variation in ctenidial mucocytes and any correlation to drifting behavior. Many questions remain on how different environmental parameters constrain the dispersal of *C. fluminea*. Additional fundamental knowledge on how temperature regulates physiological and behavioural changes in the clams with consequences in dispersal ability has been demonstrated in the present study. The results indicate that increasing temperature at high-ranges can lead to population expansion in *C. fluminea* and might be a proxy that helps predict changes in dispersal patterns correlated with change in water temperature.
